# Differential Sensitization of Muscle versus Fascia in Individuals with Low Back Pain

**DOI:** 10.3390/bioengineering9090440

**Published:** 2022-09-05

**Authors:** Ronen Cozacov, Amir Minerbi, May Haddad, Simon Vulfsons

**Affiliations:** 1 Institute for Pain Medicine, Rambam Health Campus, P.O. Box 9602, Haifa 31096, Israel; 2 Faculty of Medicine, Technion Israel Institute of Technology, P.O. Box 9602, Haifa 31096, Israel

**Keywords:** fascia, myofascial pain, pain sensitization

## Abstract

Muscles and the deep fascia surrounding them have been suggested to play an important role in various musculoskeletal pain conditions including low back pain. Both have been shown to host rich nociceptive innervation and to undergo changes in individuals with chronic pain. However, evidence for the respective contribution of muscle and fascia sensitization in humans with myofascial pain syndrome is lacking. Here, we studied the sensitization of muscle and fascia in individuals with myofascial low back pain. Twenty individuals with acute (5) and chronic (15) myofascial low back pain of the quadratus lumborum muscle and a matched control group of twenty healthy individuals were recruited and clinically evaluated. All participants underwent ultrasound-guided needling of their subcutaneous tissue, deep fascia and quadratus lumborum muscle. Reported pain intensity and episodes of muscle twitching were recorded and analyzed. Among pain patients, both muscles and deep fascia demonstrated pain hypersensitivity, but muscles were significantly more sensitized than the deep fascia. No difference between acute- or chronic-pain patients was observed. Results of this study suggest that while both deep fascia and muscle show pain sensitization in both early and chronic stages of low back pain, muscles are more sensitized than fascia.

## 1. Introduction

Myofascial pain is widely considered a common cause of pain and disability [1,2,3], with putative contribution to many clinical pain syndromes, including low back pain [4], temporomandibular pain [5,6,7], pelvic pain [8,9,10] and others. The term *myofascial pain* [11] suggests the involvement of both muscle and fascia in the pathogenesis of the syndrome. Indeed, many studies focused on the contribution of muscles to myofascial pain syndrome, demonstrating specific changes in involved muscles, including taut bands, trigger points and typical pain referral patterns [12,13]. Consequently, treatment modalities for myofascial pain have largely focused on muscles, including muscle injections [14], manual therapy [8,15], dry needling [16,17] and others [13]. Previously, emphasis was given to the role of the muscles in myofascial pain, however, in recent years, the role of fascia has drawn attention [18,19,20]. Anatomical studies demonstrate physiological features of the deep fascia, allowing it to play important roles in proprioception, force transmission and nociception [19,21]. These findings are further corroborated by studies reporting alterations in the deep fascia in individuals with chronic myofascial pain. For example, the deep fascia has been shown to contain free nerve endings [22], and their concentration in the deep palmar fascia is increased in individuals with chronic inflammation and pain [23]. In individuals with low back pain, the thoracolumbar fascia demonstrated decreased shear strain [24]. Finally, experimental sensitization of the deep fascia induces both spontaneous pain and pain hypersensitivity in humans and in animal models. Schindler et al. injected hypertonic saline into the thoracolumbar fascia, the erector spinae muscles and the subcutaneous tissues of healthy individuals [25]. They found that the injection of hypertonic saline into the deep fascia induced a longer pain duration and higher peak pain as compared with injection of the muscle and subcutaneous tissues. A recent study demonstrated similar results [26]. Hoheisel et al. used a rat model of chemical sensitization of the thoracolumbar fascia and demonstrated increased concentration of nociceptor fibers in the fascia [27] and increased dorsal horn nociceptive activity [25] following chemical sensitization.

Taken together, these data suggest that both muscles and fascia are: (1) capable of transmitting nociceptive input; (2) may demonstrate pain hypersensitization in both humans and animal models; and (3) show structural and physiological changes in humans with chronic regional pain. However, evidence for the respective contribution of muscle and fascia sensitization to myofascial pain syndrome in humans is lacking.

This study aims to investigate pain sensitization of muscle and fascia in individuals with myofascial low back pain as compared with that of healthy controls.

## 2. Methods

### 2.1. Study Design and Oversight

This study was conducted at the Rambam Institute for Pain Medicine, Haifa, Israel. The study was approved by the Rambam institutional review board (approval number 412-18 RMB).

### 2.2. Participants Recruitment and Clinical Evaluation

Patients suffering from acute or chronic low back pain (LBP) were referred by their treating physician. Healthy individuals were recruited by advertisement in the local media. Candidates underwent a thorough clinical evaluation and provided informed consent. All participants were adult (>18 years old) women and men capable of giving informed consent in Hebrew. Inclusion criteria in the LBP group were: (1) acute (<3 months) or chronic (>3 months) unilateral LBP; (2) self-reported pain intensity ≥30 (on a 0–100 numeric rating scale); and (3) history and physical findings compatible with LBP of myofascial origin attributable to the quadratus lumborum muscle. Inclusion criteria in the healthy controls group were: the absence of low back pain symptoms in the preceding 3 months.

Exclusion criteria for both groups were: (1) any active chronic pain disorder, other than LBP in the patient group (e.g., migraine, neuropathic pain and nociplastic pain); (2) woman of childbearing age; (3) individuals with a known coagulopathy or those receiving anti-coagulant/anti-platelet therapy other than aspirin; and (4) any known muscle pathology.

All participants were examined by one certified pain physician (RC) experienced in the diagnosis of myofascial pain, and the mechanism of LBP was evaluated. Participants’ demographic characteristics were documented, as well as the duration, laterality and intensity of their pain. The diagnosis of myofascial pain of the quadratus lumborum was based on the following findings [3]: (1) pain of mechanical nature; (2) deep palpation of the quadratus lumborum eliciting a characteristic tenderness, with or without referred pain; (3) recognition of the evoked pain by the patient upon palpation of the muscle tender spot; (4) limited range of motion of lower back with worsening of the pain on contralateral side flexion; and (5) no evidence upon physical examination suggestive of pain of radicular, facet or sacroiliac joint origin.

### 2.3. Ultrasound-Guided Dry Needling of the Muscle, Fascia and Skin

**Figure 1:**
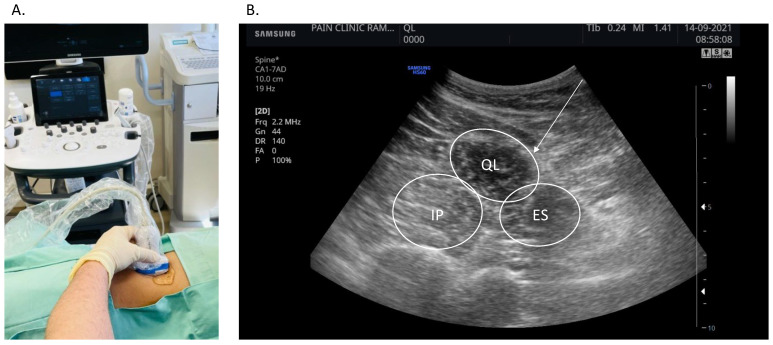
Ultrasound imaging and needling of the quadratus lumborum muscle. (**A**). Experimental setup demonstrating patient positioning in the lateral decubitus position and transducer placement. (**B**). A short-axis view of the quadratus lumborum (QL), iliopsoas (IP) and erector spinae (ES) in a healthy individual. The needle trajectory is highlighted. For clarity purposes a short-axis view is presented here; however, needling of the muscle was performed in long-axis.

All participants underwent deep dry needling of the quadratus lumborum muscle. LBP patients underwent needling in the affected quadratus lumborum muscle, while in healthy controls the side was preassigned by random allocation. With patient in the lateral decubitus position, the skin was prepped with chlorhexidine and draped. Under sterile conditions, a curved array ultrasound (US) probe (CA1-7AD-1-7 MHz, HS60, SAMSUNG) was used to visualize the long axis of the affected quadratus lumborum muscle (Figure 1). Once a satisfactory view was obtained, a 75 mm long, 0.3 mm needle (Eacu, 0.30 × 75, Maanshan Bond Medical Instruments CO., LTD, Ma’anshan, China) was advanced in plane with the US transducer towards the muscle. After subcutaneous penetration, the needle was advanced towards the deep fascia, bending it but not penetrating into the muscle. The needle was then inserted through the fascia into the muscle (Supplementary Video S1).

Participants were asked to rate their pain intensity during the procedure at three time points: when needling the subcutaneous tissue, when the needle came in touch with the fascia surrounding the quadratus lumborum muscle (without penetrating it) and while needling the muscle. For each participant, needling was repeated three times at different sites along the muscle belly, and the average pain, reported during the repetitions, was recorded on a numeric rating scale (NRS) of 0–100. The occurrence of twitches (involuntary muscle jerks), often accompanying needling of MFP affected muscles, was recorded. Participants were blinded to the depth of needle insertion.

### 2.4. Data Analysis

Sample size was calculated based on an estimated baseline pain intensity (NRS) of 70 (±17 STD) [28], allowing the detection of a 15-point within-group (fascia to muscle) and between-group (LBP to heathy controls) difference with an alpha of 0.05 and a power of 0.8. The required sample size, corrected for a 30% drop-out rate, was estimated at 20 patients per arm. Demographics, anthropometrics and clinical measures were evaluated for normality using the Wilks–Shapiro test. Normally distributed measures were compared using ANOVA and post hoc Games–Howell test. Non-normally distributed variables were compared using the non-parametric Kruskal–Wallis test and adjusted for multiple comparisons. Dichotomous variables were compared using Fisher’s Exact test. Analysis was performed on IBM SPSS^®^ Statistics, Armonk, NY, USA, version 23.

## 3. Results

### 3.1. Participant Baseline Characteristics

Forty participants were recruited, of which 20 were diagnosed with myofascial LBP and 20 were healthy controls. Of the LBP patients, 15 reported chronic pain (>3 months) and 5 reported acute pain. Participants were predominantly males (93%), 49.9 (SD 13.6) years old on average, with a mean body-mass index of 28.6 (SD 3.6) kg/m^2^. Mean pain duration among acute and chronic LBP patients was 27 and 468 days, respectively, and mean pain intensities were 53 and 46 (NRS), respectively. The demographic and anthropometric characteristics of participants in the two study groups were comparable (Table 1).

### 3.2. Evoked Pain and Twitch Response on Stimulation of the Deep Fascia, Muscle and Subcutaneous Tissues

When comparing pain intensity reported by participants while needling the subcutaneous tissue, deep fascia and muscle, pain intensities demonstrated a non-normal distribution pattern (Shapiro–Wilk *p* < 0.01); thus, the non-parametric Kruskal–Wallis test was used for comparisons. Within-group analysis demonstrated that among acute and chronic LBP patients, the needling of the fascia resulted in higher pain intensities as compared with the subcutaneous tissues (adjusted *p* = 0.011) but lower pain as compared with the needling of the muscle (adjusted *p* = 0.003). Among healthy controls, both fascia and muscle were slightly more sensitive than the subcutaneous tissues (adjusted *p* = 0.02), but no difference was observed between pain intensities in the deep fascia and in the muscle (adjusted *p* = 1).

**Figure 2:**
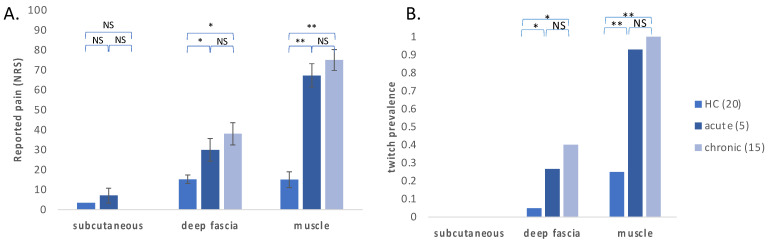
Evoked pain intensity and twitch response among LBP patients and healthy controls. (**A**) Patient-reported evoked pain in muscle, fascia and skin among LBP patients and healthy controls; (**B**) incidence of twitch response when stimulating the muscle, fascia and skin of LBP patients and healthy controls. (* *p* < 0.05 and ** *p* < 0.0001; NRS—numeric rating scale, NS—not significant).

Between-group analysis showed significant differences in the sensitization of muscle and deep fascia between acute and chronic pain patients vs. healthy controls but not between acute vs. chronic patients (Figure 2A). Among healthy controls, the needling of the deep fascia or the muscle resulted in roughly the same pain intensity, significantly lower than that reported by LBP patients. The needling of the subcutaneous tissues resulted in almost no pain among both LBP patients and healthy controls.

Thirty muscle twitches were detected, of which 24 were observed in LBP patients and 6 in healthy controls. Among LBP patients, twitches were most commonly observed during the needling of the muscle (95%) and less when needling the fascia (30%). The incidence of twitches among healthy controls was significantly lower, with observed twitches in 25% of muscle needling and 5% of fascia needling. No twitches were observed while needling the subcutaneous tissues either in LBP patients or in healthy controls.

## 4. Discussion

In this observational controlled study, we used ultrasound-guided needling to evaluate the pain evoked by three tissue types—muscle, fascia and subcutaneous tissues—in patients with myofascial pain of the quadratus lumborum and in a matched cohort of healthy controls. Evoked pain intensity was significantly higher in muscle and deep fascia among LBP patients when compared with healthy controls, regardless of pain duration. Among LBP patients, stimulation of the muscle provoked the highest pain intensities, followed by the fascia (mild pain) and then the subcutaneous tissue (mild to no pain). In contrast, among healthy controls, pain intensity upon stimulation of the muscle and fascia was comparably mild. Similarly, muscle twitches were evoked mostly in LBP patients and during muscle stimulation more than during stimulation of the fascia.

Acute and chronic LBP may differ in their underlying mechanisms, and thus, sensitization of muscle, deep fascia and subcutaneous tissues may follow different timelines. Thus, tissue sensitization was evaluated both in chronic LBP patients and in a smaller group of individuals with acute LBP. Taken together, these results suggest a differential sensitization of nociceptive pathways in patients with acute and chronic myofascial LBP, whereby muscles are sensitized more than fascia, which in turn is sensitized more than the subcutaneous tissue. Both muscle and deep fascia are more sensitized in patients (acute or chronic) as compared with healthy controls. This differential sensitization is detectable in as little as a few days after the onset of LBP and seems to persist over the course of many months.

The mechanisms underlying the selective sensitization of muscles in patients with myofascial pain remain unclear. Putative mechanisms may involve differences in the peripheral characteristics of the two tissues (e.g., nociceptor innervation density, shear tissue mass) or upstream sensitization (e.g., the sensitization of spinal cord nociceptive neurons). Studies in mice have demonstrated that the thoracolumbar fascia contains roughly the same density of free nerve endings as the back muscles, but a larger proportion of CGRP-expressing fibers [29], an observation that weakens the hypothesis that innervation density may explain increased muscle sensitization. Another mechanistic question is whether sensitization drives LBP or whether LBP induces sensitization. While this study was not designed to answer such questions, the observation that differential sensitization is detectable in patients with new-onset LBP (as short as several days) hints that muscle sensitization could play a role in LBP.

It should be stressed that the results of this study do not infer the origin of pain in myofascial pain and do not hint at a more central role of muscle as compared with fascia. Nevertheless, they do provide evidence of a differential sensitization of muscles as compared with fascia.

This study is unique in using needling to investigate muscle sensitization. Previous studies have used pressure algometry [30,31,32] or the injection of irritating substances into muscles and deep fascia [25,33,34]. Here, needling was chosen as a model of evoked mechanical pain as it allows the selective stimulation of fascia vs. muscle, without introducing sensitizing exogenous substances. Needling has been shown to evoke pain via both c-fiber and Aδ [35].

Previous studies investigating the differential effect of hypertonic saline injection into the deep fascia vs. muscles demonstrated that, in the short term, fascia sensitization resulted in significant increases in pain in the area under the curve, which was attributable mostly to longer pain duration and less to higher pain intensity [25,26]. However, these studies were conducted in healthy individuals, and thus sensitization of fascia and muscle in myofascial pain patients cannot be inferred.

The lack of subcutaneous pain sensitization may seem surprising, especially in light of previous reports of decreased pain-pressure thresholds (PPT) in individuals with LBP. In a study comparing PPT in 30 women with LBP and a group of matched healthy controls, PPT values were significantly lower in the patient group [36]. More recently, however, a study on 100 participants, including individuals with persistent LBP, episodic LBP and healthy controls, reported no significant between-group differences in PPT [37]. It is important to note, however, that the pressure thresholds reported in these studies (in the magnitude of 5–7 lbs/cm^2^) correspond not only to the skin and subcutaneous tissues but inevitably also stimulate the deep fascia and muscles. Finally, in a study exploring the association of muscle stiffness, PPT and heat pain thresholds in a cohort of 132 individuals with persistent LBP, muscle stiffness was associated with PPT but not with heat pain threshold [38], strengthening the notion that in LBP, sensitization of deep tissues (muscle and fascia) it is more prominent than that of subcutaneous tissues.

Results of this study are strengthened by several factors: The use of high-resolution US guidance allowed for an accurate evaluation of needle position and contact with the target organs. The use of multiple control variables, namely LBP patients vs. healthy individuals, and three tissue types allowed for multiple comparisons to be made. Finally, the use of several replications—20 patients per arm and three repetitions per patient—allowed a statistical power that yielded highly significant results.

This study should be viewed in light of several limitations: (1) Partial blinding—while patients were blinded to the depth of needling, the examiner was not blinded to the patient group nor to the depth of needling, thus creating a potential bias; and (2) Sample size was limited, especially in the acute LBP group. Nevertheless, results remained highly significant both between-group and within-group given the large effect size. This suggests that differential sensitization of muscle and fascia are pertinent in both acute and chronic low back pain. (3) Participants in this study were mostly men, limiting the generalizability of these results to women. (4) While needling the muscle, the needle also stimulated the fascia encapsulating it, raising the possibility that the higher pain intensity observed during muscle stimulation could in fact be attributed to pain elicited by both fascia and muscle. Nevertheless, in healthy individuals, the needling of muscle and fascia elicited comparable pain, strengthening the notion that increased pain intensity in the muscles of LBP patients represent the differential sensitization of muscle as compared with fascia. (5) Data regarding the pharmacotherapy of the study participants was not collected.

In summary, results of this study demonstrate a significant sensitization of muscle and fascia among individuals with acute and chronic myofascial LBP. Furthermore, sensitization seems predominant in the muscle, less so in the fascia and insignificant in the skin. Future studies are warranted to establish this observation in other muscles and to explore the differential contribution of muscle versus fascia in the pathophysiology of myofascial pain.

## Figures and Tables

**Table 1:** Demographic and baseline pain characteristics of study participants.

	Healthy Controls (20)	Acute Pain (5)	Chronic Pain (15)
age (years)	44.20 (12.01)	49.87 (13.58)	39.40 (16.83)
body-mass index	26.00 (3.68)	28.60 (3.56)	24.51 (3.45)
gender (% males)	0.95	0.93	0.80
baseline pain (NRS)	0.00 (0.00) *	53.33 (12.20)	46.00 (5.48)
pain duration (days)	0.00 (0.00) *	27.13 (23.34)	468.00 (272.98)

* Mean (SD) * “Healthy controls” are different from “Acute pain” and “Chronic pain”, *p* < 0.01 ANOVA and Games–Howell post hoc test.

## Data Availability

The data supporting the findings of this study are available from the corresponding author upon reasonable request.

## Supplementary Materials

The following supporting information can be downloaded at: https://www.mdpi.com/article/10.3390/bioengineering9090440/s1, Video S1: Twitch responses while needling the quadratus lumborum muscle. Ultrasound video showing a short-axis view of the quadratus lumborum muscle. A needle is introduced, and two vigorous twitch responses are observed (on seconds 11 and 21).
